# MINDEC-An Enhanced Negative Depletion Strategy for Circulating Tumour Cell Enrichment

**DOI:** 10.1038/srep28929

**Published:** 2016-07-19

**Authors:** Morten Lapin, Kjersti Tjensvoll, Satu Oltedal, Tove Buhl, Bjørnar Gilje, Rune Smaaland, Oddmund Nordgård

**Affiliations:** 1 Department of Haematology and Oncology, Stavanger University Hospital, N-4068 Stavanger, Norway; 2 Laboratory for Molecular Biology, Stavanger University Hospital, N-4068 Stavanger, Norway.; 3 Department of Mathematics and Natural Sciences, University of Stavanger, N-4036 Stavanger, Norway

## Abstract

Most current methods of circulating tumour cell (CTC) enrichment target the epithelial protein EpCAM, which is commonly expressed in adenocarcinoma cells. However, such methods will not recover the fraction of CTCs that have a non-epithelial phenotype due to epithelial–mesenchymal transition. For phenotype-independent CTC enrichment, we developed a new enhanced negative depletion strategy—termed MINDEC—that is based on multi-marker (CD45, CD16, CD19, CD163, and CD235a/GYPA) depletion of blood cells rather than targeted enrichment of CTCs. Here we validated the performance of MINDEC using epithelial and mesenchymal cancer cell lines, demonstrating a mean recovery of 82 ± 10%, high depletion (437 ± 350 residual white blood cells (WBCs)/mL peripheral blood), linearity between spiked and recovered cells (correlation coefficient: *r* = 0.995), and a low detection limit (≥1 cell recovered in all four replicates spiked with 3 cells). For clinical validation of this method, we enumerated CTCs in peripheral blood samples from patients with metastatic pancreatic cancer, detecting CTCs in 15 of 21 blood samples (71%) from 9 patients. The promising performance of the MINDEC enrichment strategy in our study encourages validation in larger clinical trials.

Circulating tumour cells (CTCs) were observed by the Australian physician Thomas Ashworth as early as 1869^1^. However, their diagnostic and prognostic potential has only recently been explored since the development of sufficiently sensitive techniques to detect these rare cells^2^. CTC involvement in the metastatic process has prompted extensive investigation during the last decade, revealing that CTCs provide clinically relevant information for a variety of cancers^3^,^4^,^5^,^6^,^7^. However, the role of CTCs in the metastatic process is not yet completely understood and technical hurdles remain^8^,^9^, and thus there is a strong need for improved methods for CTC isolation and characterization.

CTCs are extremely rare, estimated to comprise only 1 cell per billion blood cells^8^; therefore, an enrichment step is required. Current enrichment methods separate CTCs from the vast number of normal blood cells based on either physical^10^,^11^,^12^,^13^ or biological properties of CTCs^14^,^15^,^16^,^17^,^18^, or a combination of the two^19^,^20^. The majority of these methods—including the FDA-approved CellSearch™ system^14^—target the epithelial cell adhesion molecule (EpCAM), which is commonly expressed on adenocarcinoma cells^21^. However, the CTC population is reportedly rather heterogeneous, with evidence of subpopulations that express various levels of epithelial and mesenchymal transcripts^22^, and of CTCs that undergo epithelial–mesenchymal transition (EMT)^23^. Recent studies also demonstrate that the presence of CTCs expressing mesenchymal markers is associated with poor prognosis^24^,^25^,^26^,^27^. Moreover, these markers have been detected in both EpCAM-positive and EpCAM-negative CTCs^26^,^28^, suggesting that current enrichment methods that only target EpCAM-positive CTCs will fail to detect certain CTC subpopulations with potential clinical value.

Several EpCAM-independent enrichment approaches have been described—including methods involving filtration^10^,^11^; microfluidic techniques^12^,^13^; microchip devices^20^; and CTC enrichment through the antibody-directed removal of blood cells^15^,^17^,^18^, commonly known as negative depletion. These enrichment methods facilitate detection of CTCs of both epithelial and mesenchymal phenotypes, and can be used to detect CTCs in cancers of non-epithelial origin, such as sarcomas^29^. However, the investigated EpCAM-independent methods still have limitations with regard to recovery, purity, throughput, and cell viability (reviewed by Gabriel *et al*.^30^).

Here we present and validate a new enhanced negative depletion strategy, coined Multi-marker Immuno-magnetic Negative Depletion Enrichment of CTCs (MINDEC). MINDEC employs superparamagnetic beads coupled with a multi-marker antibody cocktail that targets a wide variety of different blood cell classes, thereby facilitating phenotype-independent CTC detection. As a proof of principle, we used this new strategy to detect potential CTCs in blood samples from patients with metastatic pancreatic cancer.

## Results

### Validation of the MINDEC strategy

**Figure 1:**
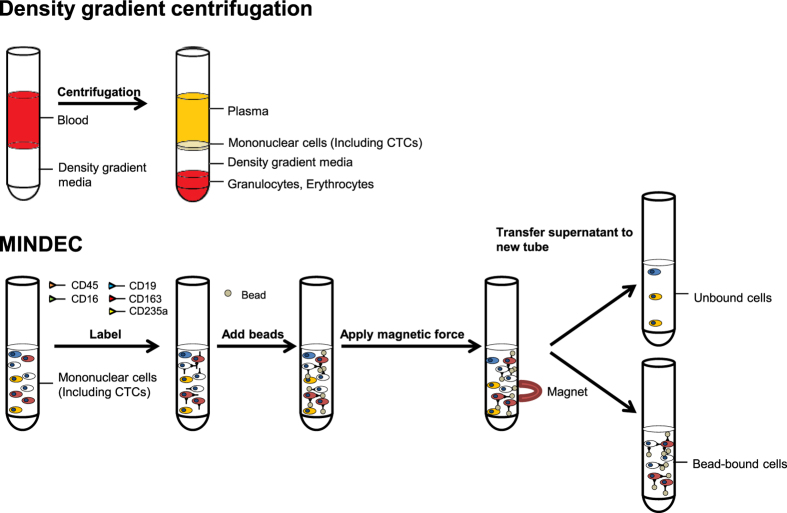
Schematic overview of the enrichment procedure. The PBMC fraction, including tumour cells, is isolated by density gradient centrifugation. This is followed by cell labelling with biotinylated antibodies against CD45, CD16, CD19, CD163, and CD235a/GYPA, and addition of streptavidin-coated superparamagnetic beads (1 μm diameter). Labelled cells bind to the superparamagnetic beads, and will be retained when magnetic force is applied. Unbound cells are recovered from the supernatant for subsequent analysis.

MINDEC, the immuno-magnetic negative depletion strategy presented in this study (Fig. 1), is an enrichment strategy targeting a wide variety of blood cells, including CD45 (pan-leucocyte), CD16 (natural killer cells and neutrophil granulocytes), CD19 (B-cells), CD163 (monocytes and macrophages), and CD235a (red blood cells; RBCs). To explore the feasibility and performance of this strategy, we validated the recovery rates for different cell lines, the enrichment efficiency, the recovery linearity, and the detection limit.

**Figure 2:**
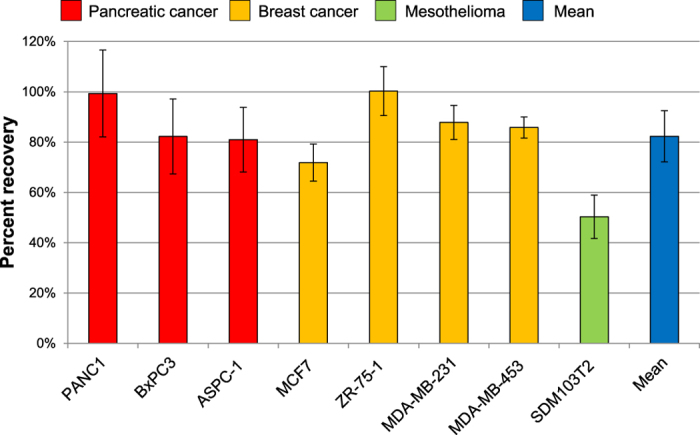
Validation of the MINDEC enrichment strategy. Average recovery rates of the different cell lines are shown, grouped by cancer of origin. The blue bar shows the average recovery of all cell lines. Error bars represent standard deviation (n = 3).

To evaluate the recovery—i.e. the number of cells detected relative to the number of cells spiked into the peripheral blood mononuclear cell (PBMC) fraction—we used 8 different cell lines: the human pancreatic cancer cell lines PANC1, BxPC3, and ASPC-1; the human breast cancer cell lines MCF7 (Luminal A), ZR-75-1 (Luminal B), MDA-MB-231 (Triple negative/basal), and MDA-MB-453 (HER2^+^); and the mesenchymal human mesothelioma cell line SDM103T2. We spiked 1000 cancer cells of each cell line into PBMC fractions obtained from 9 mL whole blood (n = 3), and enriched each sample using MINDEC. Using flow cytometry, we measured the recovery of the spiked cell line cells—which ranged from 50 ± 9% for SDM103T2 to 100 ± 10% for the epithelial breast cancer cell line ZR-75-1, with a mean recovery of 82 ± 10% across all eight cell lines (Fig. 2).

In parallel with the recovery experiments, we also recorded the enrichment efficiency, i.e. the proportion of CD45^+^ white blood cells (WBCs) depleted in the enrichment. When using MINDEC we achieved an average of 3-log depletion of the pre-enriched WBCs. Combined with the WBCs depleted in the density gradient centrifugation pre-enrichment step, this resulted in a mean residual leucocyte count of 437 ± 350 WBCs/mL peripheral blood (range: 98–1533 WBCs/mL peripheral blood). The blood donor was the single most variable factor influencing the depletion of WBCs.

**Figure 3:**
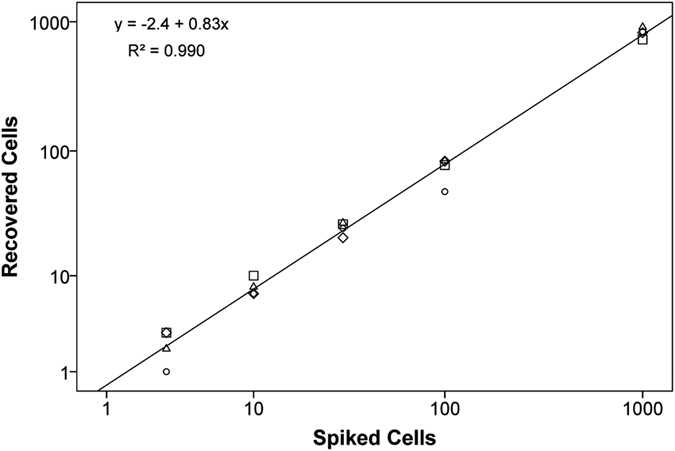
Recovered numbers of ZR-75-1 cells plotted against spiked numbers. The plot includes a linear regression line and the associated regression coefficients (upper left corner). Each replicate series (n = 4) is distinguished with separate markers.

To evaluate the recovery linearity and to determine the detection limit of the MINDEC strategy, we spiked increasing numbers of ZR-75-1 cells (0, 3, 10, 30, 100, and 1000 cells) into 5 × 10^6^ PBMCs. To determine linearity, the recovered cells were plotted against the initial numbers of spiked cells (Fig. 3). Linear regression yielded a slope of 0.83, with excellent linearity between the spiked and recovered cell numbers indicated by a Pearson’s correlation coefficient (r) of 0.995. Among the different spiking levels, the mean recovery rates varied from 73–82%, with a higher coefficient of variation for lower numbers of spiked cells—ranging from 43% for the samples spiked with 3 cells to 9% for the samples spiked with 1000 cells. The detection limit was very low, with at least 1 cell recovered in all four samples spiked with 3 cells.

### Cancer cell recovery after density gradient centrifugation

To estimate the amount of cells lost during the pre-enrichment step using Lymphoprep™, we compared recovery after the MINDEC procedure between cells spiked into whole blood vs. spiked into the PBMC fraction after density gradient centrifugation. The recovery in the samples spiked into whole blood before density gradient centrifugation was only 65 ± 16% (n = 4) compared to 87 ± 11% (n = 4) in the samples spiked into the PBMC fraction after density gradient centrifugation, providing an estimate of CTC loss during the pre-enrichment step.

### CTC detection in patient samples

As a proof of principle, we processed 21 blood samples from 9 patients with metastatic pancreatic cancer by density gradient centrifugation and used our validated MINDEC enrichment strategy to detect potential CTCs. Blood samples were drawn at different time-points during the disease course from patients undergoing chemotherapy.

**Figure 4:**
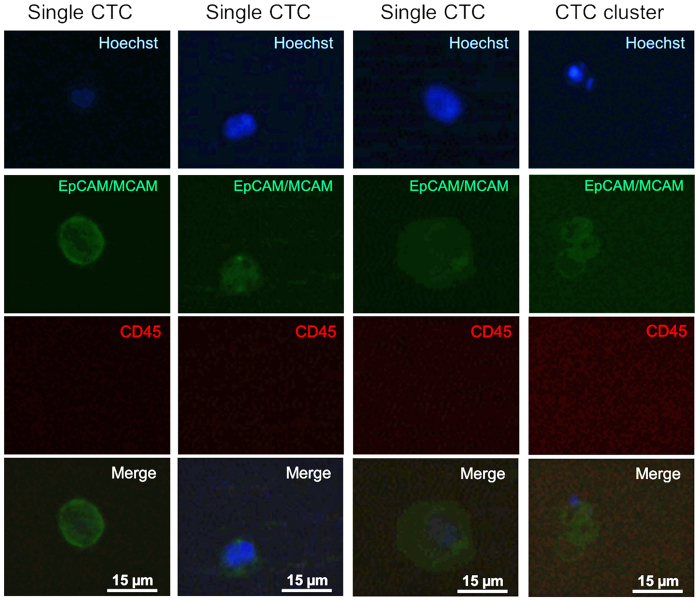
Representative fluorescence images of CTCs and a CTC cluster isolated from peripheral blood of patients with metastatic pancreatic cancer. Enriched blood samples were stained with Hoechst 33343 (nuclei, blue), EpCAM (green), MCAM (green), and CD45 (red).

EpCAM-MCAM^+^/CD45^−^ CTCs were detected in 15 of the 21 processed patient blood samples (71%), with a mean of 2 ± 2 CTCs/9 mL blood. Among the nine included patients, seven had detectable CTCs in at least one blood sample. The CTCs detected in the patient samples varied in size, ranging from 5 μm to over 20 μm (Fig. 4 and Supplemental Figure S1), in accordance with previously reported data^31^. We also detected apparent CTC clusters in 3 of the 21 patient samples (14%). Two of these samples originated from the same patient and contained homotypic clusters comprising only CTCs, including one containing three CTCs (Fig. 4). In the sample from another patient, we found a heterotypic cluster comprising one CTC and one CD45^+^ WBC (Supplemental Fig. S1). Both patients with detectable CTC clusters experienced disease progression at the times of cluster detection.

## Discussion

Several unique properties of CTCs (size, density, protein expression, electric charges, and deformability) offer possibilities to distinguish them from hematopoietic cells; however, their heterogeneity can complicate CTC enrichment. Methods that enrich CTCs using anti-EpCAM antibodies (such as the CellSearch^®^ system^14^) may fail to enrich CTCs that have a mesenchymal phenotype and lack epithelial protein expression. Additionally, enrichment methods based on physical properties, such as filtration and microfluidic methods, will likely fail to recover smaller CTCs. Filtration methods also have issues with low purity, sample clogging, and cell damage^30^. As an alternative, negative depletion strategies have been employed in which hematopoietic cells are removed by anti-CD45 antibodies^17^,^18^. However, using CD45 to deplete cells leaves large amounts of contaminating WBCs (0.8–1.2 × 10^4^ WBCs/mL peripheral blood) after enrichment^17^. The RosetteSep™ CTC Enrichment Cocktail—which targets CD2, CD16, CD19, CD38, CD45, CD66b, GYPA, and either CD36 or CD56 depending on tumour origin—is also currently on the market^32^,^33^. However, this method shows a rather low recovery of epithelial cell line cells (~60%)^15^, suggesting that this cocktail may target too many surface proteins to be truly non-specific to the CTC population. Furthermore, the depletion rate reported by the manufacturer for this cocktail shows considerably variation, from 3.2 to 4.7 log depletion^32^,^33^. Our MINDEC strategy includes a specific combination of hematopoietic markers that considerably improved WBC depletion compared to with CD45 alone, while still being able to recover both epithelial and mesenchymal CTCs, CTCs of various sizes, and CTC clusters.

Our experiments to validate the MINDEC strategy demonstrated a mean tumour cell recovery of 82 ± 10% (range, 50–100%) across the 8 evaluated cell lines, with linear recovery across the entire spiking range (from 3–1000 cells). Mean recovery rates with the CellSearch^®^ system, the Human CD45 Depletion Kit (Easysep™, STEMCELL™ Technologies), and the CTC-iChip^neg^ have been estimated as >85%^31^, 58%^17^, and 97 ± 2.7%^20^, respectively. As our results showed rather large differences in recovery among the different cell lines, the recovery estimates are not necessarily comparable with prior data using different cell lines for validation. Moreover, enrichment methods with high depletion of hematopoietic cells may also remove cells of interest due to non-specific enrichment, resulting in lower overall CTC recovery. In our case, we achieved high enrichment efficiency—with residual WBC counts of 437 ± 350/mL peripheral blood—without compromising the recovery. Our method also showed a low detection limit, with ≥1 cell detected in all four samples spiked with 3 cells. Thus, our strategy may facilitate CTC detection even in patient samples with low numbers of CTCs.

Interestingly, our recovery of the cell line MDA-MB-231 was the second highest among the evaluated breast cancer cell lines. This cell line has a fibroblast-like appearance and has previously been defined as a mesenchymal-like cell line^34^, suggesting that our enrichment strategy successfully recovers CTCs with a mesenchymal phenotype. On the other hand, recovery of the mesenchymal cell line SDM103T2 was low compared to epithelial cell lines, possibly due to hematopoietic marker expression in SDM103T2. Interestingly, the EpCAM expression detected by flow cytometry in the different cell lines (Supplemental Fig. S2) showed no relationship between EpCAM expression and recovery rate. In fact, the two cell lines with the highest recovery rates, PANC1 and ZR-75-1, had low and high EpCAM expression, respectively. Thus, as expected, the tumour cell recovery with our enrichment strategy did not seem to be dependent on EpCAM expression level.

One drawback of the MINDEC strategy is that it requires a pre-enrichment step to remove contaminating RBCs and platelets. Here we used Lymphoprep^®^ density gradient media for this purpose, which also depletes the sample of granulocytes. In the samples enriched with the MINDEC strategy, recovery was 25% lower when tumour cells were spiked before density gradient centrifugation compared to when tumour cells were spiked after density gradient centrifugation. Thus, this loss was higher than the loss estimated for the downstream MINDEC step. For future studies, RBCs and platelets may be removed using microfluidics^12^,^35^, which enables low-shear separation of nucleated cells from RBCs and platelets with minimal loss of nucleated cells^12^, although smaller CTCs may be lost using these methods^30^. Microfluidics can also be combined with immuno-magnetic enrichment in a single device, as demonstrated with the CTC-iChip^20^.

In our present study, we used the mesenchymal marker MCAM^36^ to detect mesenchymal CTCs. In the CellSearch^®^ Endothelial Cell Kit^37^, MCAM is used to enrich circulating endothelial cells (CECs), although MCAM is reportedly expressed on vascular endothelium rather than on CECs^38^. Since CECs are present in both healthy individuals and diseased patients^37^, any CECs enriched by MINDEC should be present in both healthy individuals and pancreatic cancer patients. Accordingly, three of the control samples included EpCAM-MCAM^+^ cells that may have been CECs; however, we could not determine whether these cells were EpCAM and/or MCAM positive. These cells constituted a homogenous population, showing a round shape and small size (~6 μm), inconsistent with previous reports of CECs exceeding 10 μm^39^. The potential CECs observed in our present study also clearly differed from the CTCs found in the patient samples, which were generally larger and had different nucleus-to-cytoplasm ratios. An experienced haematologist concluded that these potential CECs were uncommon in peripheral blood, and were likely megakaryocyte fragments with intact DNA. This suggests that potential CECs present in the blood samples were either removed during the MINDEC procedure or were not detected by the MCAM marker used in this study.

In our proof-of-principle experiment, we detected CTCs in 71% of the blood samples from patients with advanced pancreatic cancer (mean 2 ± 2 CTCs/9 mL blood). Prior studies using the Cellsearch^®^ system to detect CTCs in pancreatic cancer have shown slightly lower positivity rates and higher mean numbers of detected CTCs, including 50% (mean: 2 ± 6 CTCs/7.5 mL blood)^31^, 42% (mean: 16.9 CTCs/7.5 mL blood, range: 0–105)^40^, 40% (mean: 6 CTCs/7.5 mL blood, range: 0–144)^41^, and 21.4% (mean: 0.7 ± 1.9 CTCs/7.5 mL blood, range: 0–7)^42^. Our higher positivity rate could be due to the higher number of metastatic patients (100%) included in our study—although in the study by Kurihara *et al*., 24/26 (92%) of the patients had metastatic disease at the time of blood sampling^40^. A more likely explanation could be the additional detection of non-epithelial CTCs in our study. Researchers using an EpCAM-independent enrichment method—Isolation by Size of Tumour cells (ISET^®^)^41^,^43^—have reported considerably higher amounts of CTCs in pancreatic cancer patients, with CTC detection in 93% (mean: 26 CTCs/7.5 mL peripheral blood, range: 0–240)^41^ and 78% (mean: 18 CTCs/mL peripheral blood, range: 0–251)^43^ of patients. The high positivity rates and the high numbers of CTCs reported in these studies may be related to the detection of CTCs using pan-cytokeratin markers, which are intracellular markers that target a wider spectrum of epithelial-specific proteins. We think that using pan-cytokeratin markers to detect CTCs would have further increased our present CTC detection rate. However, since we wanted to isolate viable CTCs from the pancreatic cancer patients for other purposes, it was not feasible to perform cell fixation and staining using pan-cytokeratin antibodies.

Recent evidence suggests that CTC clusters possess increased metastatic potential compared to single CTCs, and that cancer patients with detectable CTC clusters show shorter survival^44^,^45^. CTC clusters also reportedly express mesenchymal markers indicative of EMT^22^. In one of our patients, two follow-up samples contained homotypic CTC clusters comprising several CTCs. In a sample from another patient, we detected a heterotypic cluster containing one CTC and one WBC, demonstrating the capacity of our method to enrich CTC clusters. Interestingly, both patients with detectable CTC clusters experienced disease progression at the times of CTC cluster detection. In another patient sample, we found a homotypic cluster comprising five cells that were positive for both EpCAM-MCAM and CD45. However, this cluster was likely a staining artefact, as WBCs are known to form cell clusters during immune cell interactions^46^.

The clinical utility of EpCAM^−^ CTCs was questioned in a recent pilot study by de Wit *et al*., where the finding of EpCAM^−^ CTCs was not associated with poor prognosis^47^. However, the EpCAM^−^ CTCs in this study were detected using pan-cytokeratin antibodies, and thus it seems like the investigators of this study have not taken into consideration the potential presence of mesenchymal CTCs in the EpCAM^−^ cell fraction. Mesenchymal CTCs may constitute large portions of the total CTC fraction^22^, and transient phenotypes that are partly epithelial and partly mesenchymal may also exist^22^,^43^. In contrast to the findings of de Wit *et al*., several studies have demonstrated that mesenchymal and transient CTC phenotypes are associated with inferior prognosis regardless of EpCAM expression^24^,^25^,^26^,^27^, thus emphasizing the advantage of EpCAM-independent CTC enrichment.

In this study, we established a new enhanced negative depletion strategy for CTC enrichment—coined MINDEC—that depletes blood samples of hematopoietic cells and recovers CTC clusters, as well as both epithelial and mesenchymal CTCs. The high sensitivity for CTCs makes this strategy useful for CTC detection in cancer patients even when these cells are especially rare, such as in early stage cancer. The high depletion of leucocytes also makes this strategy useful for downstream analyses where low background levels of leucocytes are required. Furthermore, due to the independence of epithelial-specific markers, this strategy could be applied to detect CTCs in all types of cancers involving CTC presence. As a proof of principle, we demonstrated the applicability of this strategy by detecting CTCs in peripheral blood from patients with pancreatic cancer, a cancer type in which CTCs are usually present in low numbers. Our initial results suggest that the MINDEC strategy for CTC enrichment warrants validation in larger clinical studies.

## Materials and Methods

### Control and patient samples

We collected peripheral blood samples (9 mL in EDTA tubes) from healthy volunteers recruited at Stavanger University Hospital, and from patients with metastatic pancreatic cancer receiving chemotherapy at Stavanger University Hospital between July 2015 and February 2016. The study included a total of 21 blood samples from 9 patients and blood samples from 13 volunteers, all of which were processed by density gradient centrifugation and the MINDEC protocol within 2 hours of collection. Treatment response was defined using standard disease evaluation by imaging, applying the RECIST 1.1 criteria^48^. All volunteers and patients provided written informed consent to participate in the study, and the project was approved by the Regional Committees for Medical and Health Research Ethics (REK-Vest 2011/475). All methods were carried out in accordance with approved guidelines.

### Density gradient centrifugation

PBMCs—including potential CTCs in patient samples—were isolated from all blood samples by density gradient centrifugation using Lymphoprep™ density gradient medium (Axis Shield, Norway) following the manufacturer’s instructions. Briefly, diluted blood samples (0.9% NaCL, 1:1) were layered over Lymphoprep™ media and centrifuged at room temperature at 800 × g for 20 min. After centrifugation, mononuclear cells were washed with 0.9% NaCl and subsequently with PBS and then centrifuged at 4 °C at 300 × g for 10 min to pellet the cells.

### MINDEC negative depletion

After density gradient centrifugation, the PBMCs were resuspended in 100 μL isolation buffer (PBS supplemented with 2 mM EDTA and 0.1% BSA). To this, we added a cocktail (15 μL) of biotinylated antibodies: 4 μL CD45 (Molecular Probes), 4 μL CD16, 2 μL CD19, 1 μL CD163, and 4 μL CD235a/GYPA (all eBioscience). This mixture was incubated for 20 min at 4 °C, and then washed with cold isolation buffer and centrifuged at 4 °C at 300 × g for 10 min to remove excess antibodies. After centrifugation, the cells were resuspended in 900 μL isolation buffer along with 100 μL washed Depletion MyOne™ SA Dynabeads^®^ (Life Technologies AS, Norway), and this mixture was incubated at 4 °C for 15 min with tilt and rotation in a HulaMixer™ (Invitrogen). We then added 1 mL isolation buffer, and the cells and beads were thoroughly mixed by pipetting. Next, the sample was exposed to a strong magnetic field (DynaMag™ 5 or DynaMag™ 15) for 3 min, and the supernatant containing recovered unbound cells was transferred to a new tube. We then repeated the addition of 1 mL isolation buffer to the beads and the 3 min magnetic separation to recover any unbound cells not recovered in the first separation. The supernatants from the two separation procedures were combined, exposed to a strong magnetic field for 3 min to remove residual beads, and centrifuged again at 4 °C at 300 × g for 10 min. All volumes in the MINDEC procedure were added per 1 × 10^7^ cells, and scaled up accordingly in samples with higher cell numbers. The tubes and solutions were kept on ice or at 4 °C throughout the procedure.

### Cell line cultivation and spiking experiments

Spiking experiments were performed using the following cell lines as models for CTCs: the human pancreatic cancer cell lines PANC1, BxPC3, and ASPC-1 (all from the European Collection of Authenticated Cell Cultures; ECACC), the human breast cancer cell lines MCF7 (Luminal A, ECACC), ZR-75-1 (Luminal B, ATCC), MDA-MB-231 (Triple negative/basal, ECACC), and MDA-MB-453 (HER2^+^, ATCC), and the mesenchymal human mesothelioma cell line SDM103T2 (ECACC). These cell lines were cultured following the manufacturer’s recommendations, except that the culture media was supplemented with 5 mL penicillin-streptomycin (Sigma-Aldrich). Cells were harvested using 0.25% trypsin/EDTA (Sigma-Aldrich) for 3–5 min at 37 °C.

To prevent cell aggregation, the harvested cells were diluted 1:10 in staining buffer (PBS supplemented with 2 mM EDTA and 0.5% BSA). The cells were counted using a haemocytometer to determine recovery. One thousand cells from each cell line were spiked into three parallel PBMC fractions (obtained from 9-mL peripheral blood samples from a healthy volunteer). To estimate recovery during our pre-enrichment step with Lymphoprep™ density gradient media, we spiked 1000 ZR-75-1 cells into four 9-mL whole blood samples, and compared these to four samples spiked into the PBMC fraction after density gradient centrifugation. For linearity determination, we prepared a dilution series containing 0, 3, 10, 30, 100, and 1000 ZR-75-1 cells. To confirm the spiking numbers of the 3- and 10-cell samples, cells in buffer were transferred to a microscopy slide for evaluation using an Olympus XI 81 inverted microscope, followed by individual cell isolation using an MMI CellEctor cell manipulator (Molecular Machines & Industries). Each cell dilution was spiked into two replicate samples containing 5 × 10^6^ PBMCs from a healthy volunteer. All spiked samples were enriched using the MINDEC strategy.

### Fluorescent labelling

Following the MINDEC procedure, enriched samples were resuspended in 100 μL cold staining buffer along with 25 μL FcR blocking reagent (Miltenyi Biotech). The samples were further processed following one of the following three procedures. Samples from recovery experiments to be enumerated by flow cytometry were incubated with 2.5 μL EpCAM-FITC (clone HEA-125, Miltenyi Biotech), 2.5 μL MCAM-FITC (clone OJ79c, AbD Serotec^®^; for cell line SDM103T2 only), and 2.5 μL CD45-APC (clone 5B1, Miltenyi Biotech). Samples from linearity experiments to be enumerated by microscopy were incubated with 2 μL EpCAM-FITC (clone HEA-125, Miltenyi Biotech), 2 μL CD45-DyLight550 (clone T29/33, Leinco Technologies, Inc.), and 2 μL Hoechst 33342 (Molecular Probes). Finally, patient samples were incubated with 2 μL EpCAM-FITC (clone HEA-125, Miltenyi Biotech), 2 μL MCAM-FITC (clone OJ79c, AbD Serotec^®^), 2 μL CD45-DyLight550 (clone T29/33, Leinco Technologies, Inc.), and 2 μL Hoechst 33342 (Molecular Probes). All samples were incubated for 20 minutes in darkness at room temperature during staining, and were subsequently resuspended in an appropriate volume of cold staining buffer (500 μL for flow cytometry experiments, 50 μL for microscopy enumeration experiments, and 150 μL for patient samples).

### Enumeration of spiked cells by flow cytometry

**Figure 5:**
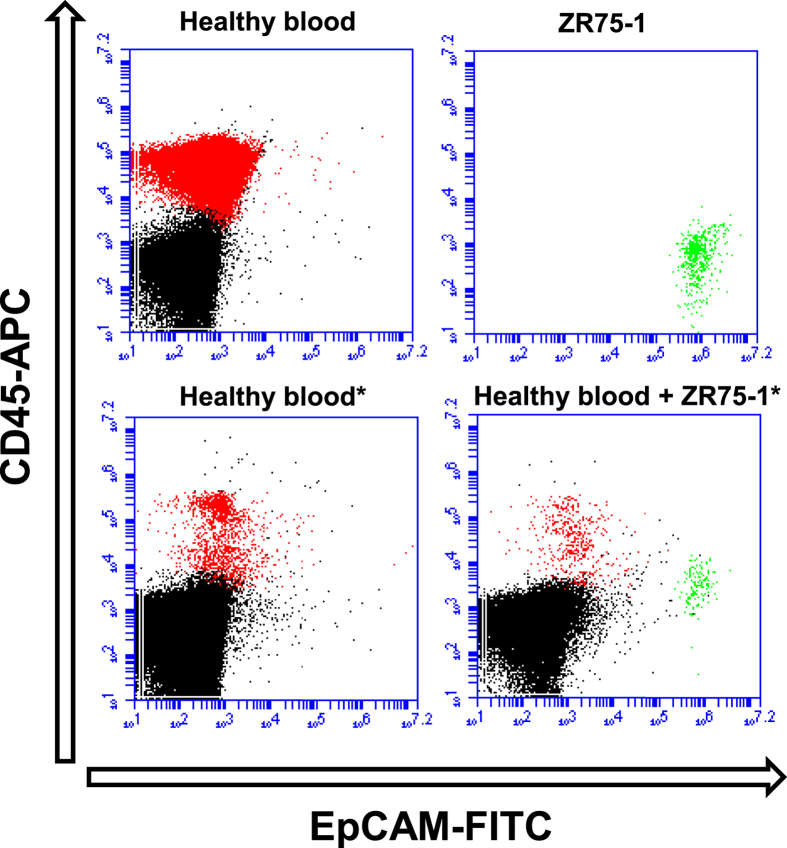
Flow cytometry analysis of PBMCs and ZR-75-1 tumour cell line cells before and after MINDEC enrichment. Top left: Blood cells from a healthy control sample after Lymphoprep^®^ density gradient separation. Top right: Pure population of ZR-75-1 cells. Bottom left: Blood cells from a healthy control sample after Lymphoprep^®^ and MINDEC enrichment. Bottom right: Blood cells from a healthy control sample spiked with 1000 ZR-75-1 cells after Lymphoprep^®^ and MINDEC enrichment. The CD45^+^ population is coloured red and the EpCAM^+^ population is coloured green. *Denotes samples enriched with MINDEC.

Flow cytometry enumeration was performed using an Accuri C6 Flow cytometer (Becton Dickinson). We used pure cell line and pure WBC populations to define EpCAM (MCAM)-positive (P1 gate) and CD45-positive cells (P2 gate), respectively, by plotting side scatter A (SSC-A) versus FITC or APC. An SSC-A versus signal width gate (P3 gate) was also defined for size-based cell sorting since cell line cells are considerably larger than WBCs. Figure 5 shows the plots of the different cell populations, and Supplemental Fig. S3 illustrates the gating strategy. Tumour cells were enumerated based on detection in both the P1 and the P3 gates. To estimate recovery, we compared the numbers of recovered spiked cells in each sample after enrichment to unstained control samples containing 10,000 cell line cells (10× the amount of spiked cells). The spiked cells and the positive control samples were both acquired from the same tumour cell line suspension. Recovery was estimated as follows:


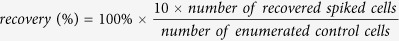


To estimate enrichment efficiency, the residual WBCs enumerated using the P2 and P3 gates were compared to an unenriched sample of PBMCs. Data acquisition and analysis were performed using BD Accuri™ C6 Software version 1.0.264.21 (Becton Dickinson).

### Microscopy enumeration of spiked cells

Enriched spiked samples were deposited on poly-L-lysine coated slides (Superfrost™ PLUS, Thermo Scientific) and left to dry overnight at room temperature in the dark. The next day, we added 20 μL mounting media (Dako, Glostrup, Denmark) to each sample, mounted the cover slips, and again left the slides to dry overnight at room temperature in darkness. The prepared slides were next transferred to an Axioplan 2 upright microscope (Zeiss, Germany) running Metafer 4 software (Metasystems, Germany), which enables automated slide scans and fluorescence detection of cells. For each slide, we acquired 3-channel fluorescent images of multiple fields covering the full area of the sample, using 10× magnification (Zeiss Plan-Apochromat 10×/0.45 M27). Exposure times were fixed at 20 ms for Hoechst 33342, 540 ms for EpCAM, and 540 ms for CD45. The fluorescent images were manually inspected to identify EpCAM^+^/Hoechst 33342^+^/CD45^−^ cells. All sample slides were reviewed by the same investigator (M.L.).

### CTC enumeration in patient samples

Enriched patient samples were transferred to a microscopy slide coated with Silanization Solution I (Sigma), and imaged using an Olympus XI 81 inverted microscope using 20× magnification (Olympus LUCPLFLN 20×). Exposure times were fixed at 86 ms for Hoechst 33342, 1400 ms for EpCAM-MCAM, and 1600 ms for CD45. Each sample was manually inspected to identify possible CTCs. Cells with a visible nucleus, no beads attached, EpCAM-MCAM expression, no CD45 expression, and a round or oval shape were enumerated as CTCs. A CTC cluster was enumerated as one cell. Due to the weak background staining of some WBCs, we established a threshold for EpCAM-MCAM positivity by processing 10 blood samples from healthy volunteers in the same manner as the patient samples. Cells in the patient samples with fluorescence emission above this threshold level were considered CTCs. All patient and control samples were reviewed by the same person (M.L.). In unclear cases, additional blood cell classification was performed by an experienced haematologist. To enhance the visibility, we adjusted the brightness and contrast of all presented microscopy images following the same procedure, with these adjustments applied to the entire images.

### Statistical analyses

All statistical analyses were performed using IBM SPSS version 22.0 (www.spss.com). Linear regression was performed and R^2^ was computed using the Linear Regression function, with number of recovered cells as the dependent variable and number of spiked cells as the independent variable. Pearson’s r was calculated using the Bivariate Correlations function. All measurements are expressed as mean ± standard deviation.

## Additional Information

**How to cite this article**: Lapin, M. *et al*. MINDEC-An Enhanced Negative Depletion Strategy for Circulating Tumour Cell Enrichment. *Sci. Rep.* **6**, 28929; doi: 10.1038/srep28929 (2016).

## Supplementary Material

Supplementary Information
