# Supplementary material for: MINDEC-An Enhanced Negative Depletion Strategy for Circulating Tumour Cell Enrichment
Source: Sci Rep. 2016 Jul 19;6:28929. doi: 10.1038/srep28929 (PMC4949475; doi:10.1038/srep28929)
Supplement: Supplementary Information [file srep28929-s1.pdf]

Supplemental Information

MINDEC - An Enhanced Negative Depletion Strategy for Circulating Tumour Cell Enrichment

Morten Lapin[1,2,3], Kjersti Tjensvoll[1,2], Satu Oltedal[1,2], Tove Buhl[1], Bjørnar Gilje[1],

Rune Smaaland[1,2] and Oddmund Nordgård[1,2]

1 Department of Haematology and Oncology, Stavanger University Hospital, N-4068 Stavanger, Norway.

2 Laboratory for Molecular Biology, Stavanger University Hospital, N-4068 Stavanger, Norway.

3 Department of Mathematics and Natural Sciences, University of Stavanger, N-4036 Stavanger, Norway.

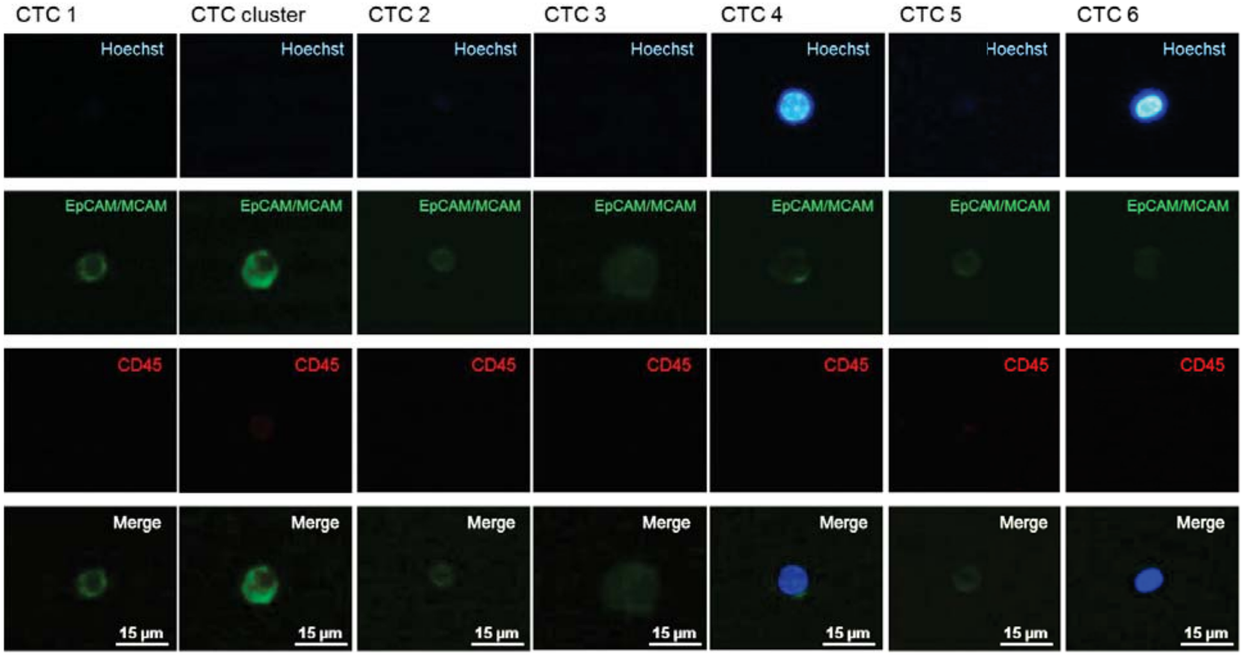

Supplemental Figure S1. All CTCs enumerated in patient sample PC18B9, showing differences in size and EpCAM-MCAM expression. The CTC cluster contains a CTC as well as a WBC expressing CD45. Cells are stained with Hoechst 33343 (nuclei, blue), EpCAM-MCAM-FITC (green), and CD45-DyLight550 (red).

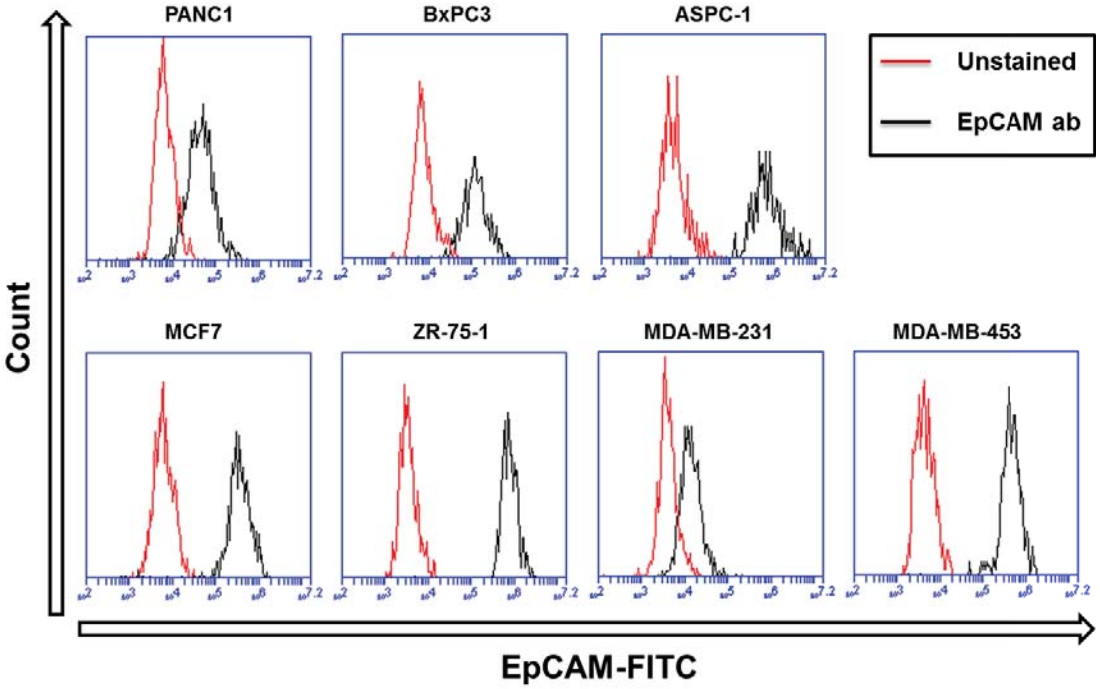

Supplemental Figure S2. Flow cytometry detection of EpCAM-FITC expression in the different cell lines used to validate the MINDEC strategy. The mesenchymal cell line SDM103T2 was not evaluated.

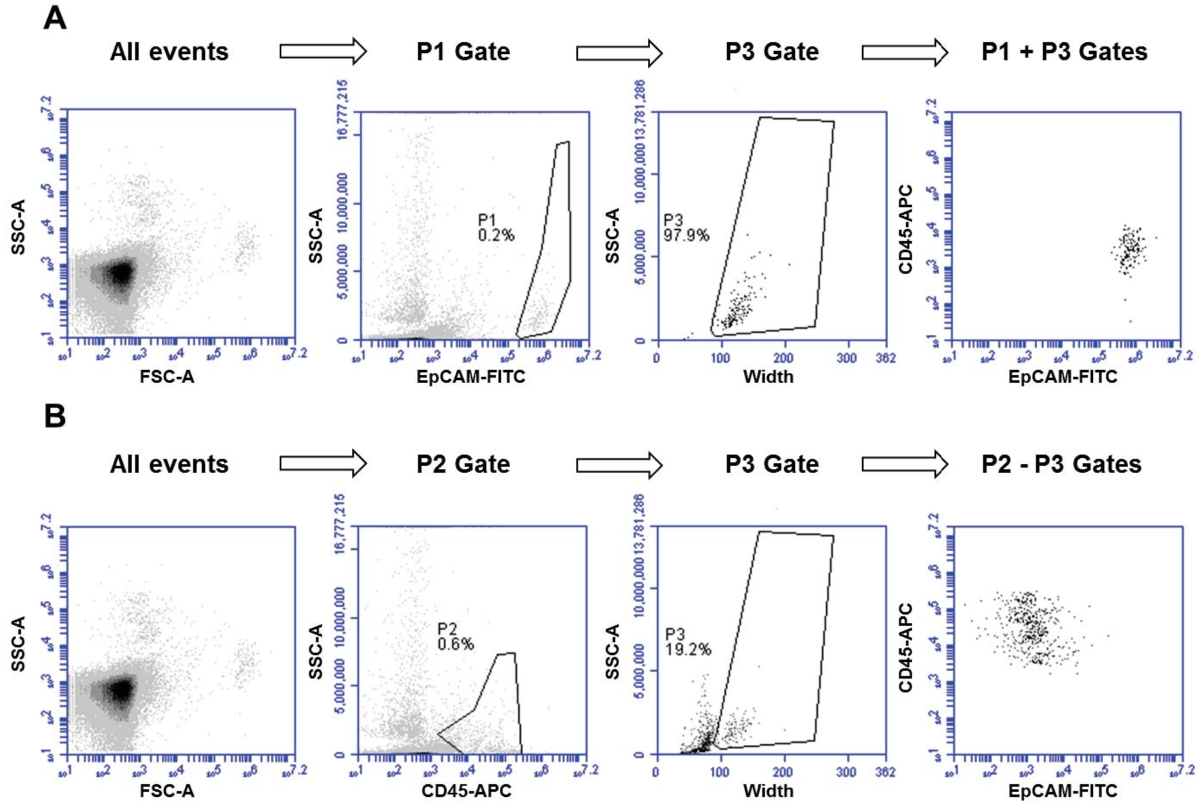

Supplemental Figure S3. Gating strategy for flow cytometry samples stained with EpCAM-FITC and CD45-APC. The first image represents blood cells from a healthy control sample spiked with 1000 ZR-75-1 cells. A) Cancer cell line cells were first gated by plotting SSC-A vs EpCAM-FITC (P1 gate); then by size gating plotting SSC-A versus signal width (P3 gate). The P1 + P3 gate plot shows the sample after gating. B) WBCs were first gated by plotting SSC-A versus CD45-APC (P2 gate), and then cells were excluded by size (P3 gate). The last plot shows sample after gating (P2 gate + removal of cells in P3 gate).
